# African Relapsing Fever Borreliae Genomospecies Revealed by Comparative Genomics

**DOI:** 10.3389/fpubh.2014.00043

**Published:** 2014-05-14

**Authors:** Haitham Elbir, Laurent Abi-Rached, Pierre Pontarotti, Niyaz Yoosuf, Michel Drancourt

**Affiliations:** ^1^URMITE, UMR63, CNRS 7278, IRD 198, INSERM 1095, Aix Marseille Université, Marseille, France; ^2^Equipe ATIP, Centre National de la Recherche Scientifique, Laboratoire d’Analyse, Topologie, Probabilités – Unité Mixte de Recherche 7353, Aix Marseille Université, Marseille, France; ^3^Equipe EBM, Centre National de la Recherche Scientifique, Laboratoire d’Analyse, Topologie, Probabilités – Unité Mixte de Recherche 7353, Aix-Marseille Université, Marseille, France

**Keywords:** *Borrelia*, relapsing fever, genomics, phylogeny, speciation

## Abstract

**Background:** Relapsing fever borreliae are vector-borne bacteria responsible for febrile infection in humans in North America, Africa, Asia, and in the Iberian Peninsula in Europe. Relapsing fever borreliae are phylogenetically closely related, yet they differ in pathogenicity and vectors. Their long-term taxonomy, based on geography and vector grouping, needs to be re-apprised in a genomic context. We therefore embarked into genomic analyses of relapsing fever borreliae, focusing on species found in Africa.

**Results:** Genome-wide phylogenetic analyses group Old World *Borrelia crocidurae, Borrelia hispanica, B. duttonii, and B. recurrentis* in one clade, and New World *Borrelia turicatae* and *Borrelia hermsii* in a second clade. Accordingly, average nucleotide identity is 99% among *B. duttonii, B. recurrentis*, and *B. crocidurae* and 96% between latter borreliae and *B. hispanica* while the similarity is 86% between Old World and New World borreliae. Comparative genomics indicates that the Old World relapsing fever *B. duttonii, B. recurrentis, B. crocidurae*, and *B. hispanica* have a 2,514-gene pan genome and a 933-gene core genome that includes 788 chromosomal and 145 plasmidic genes. Analyzing the role that natural selection has played in the evolution of Old World borreliae species revealed that 55 loci were under positive diversifying selection, including loci coding for membrane, flagellar, and chemotaxis proteins, three categories associated with adaption to specific niches.

**Conclusion:** Genomic analyses led to a reappraisal of the taxonomy of relapsing fever borreliae in Africa. These analyses suggest that *B. crocidurae, B. duttonii*, and *B. recurrentis* are ecotypes of a unique genomospecies, while *B. hispanica* is a distinct species.

## Introduction

Among spirochetes, the genus *Borrelia* includes the Lyme disease group of borreliae in North America and Europe and the relapsing fever group of borreliae in North America, Africa, Asia, and in the Iberian Peninsula in Europe (1). Six relapsing fever species have representatives that have been cultured, whereas three uncultured species are detected on the basis of original sequences (2). Eight of the species defined by sequence analysis are found in Africa, including the four definite human pathogens *Borrelia hispanica, B. crocidurae, B. duttonii*, and *B. recurrentis*. In Africa, these relapsing fever borreliae are transmitted by *Ornithodoros* ticks and the *Pediculus humanus* body louse (3–7).

Although relapsing fever borreliae are genetically very similar, they differ in vector, host range, and disease spectrum. *B. recurrentis* is a host-restricted species that is documented in human and human lice, most commonly producing deadly infections if left untreated (7). *B. crocidurae* is found in humans and in several rodent species, causing a febrile tick-borne infection less severe than the louse-borne infection in humans (8). Finally, *B. duttonii* has a narrow host range and is associated with mortality and pregnancy loss (9).

Relapsing fever borreliae were initially classified on the basis of vector and geography (1). However, the coexistence of several species in the same geographic area such as *B. hispanica* and *B. crocidurae* in North Africa (10, 11) and *B. duttonii* and an unnamed *Borrelia* sp. (AB105117) (12) in Tanzania questioned this conventional classification. In addition, 16S rRNA gene sequence similarity between such defined species was above the borderline that typically distinguishes other bacterial species (13).

Therefore, revisiting the long-term taxonomy of relapsing fever borreliae in Africa is needed. The recent availability of genome sequences for *B. crocidurae, B. duttonii, B. recurrentis, B. hispanica* (14–16), *B. turicatae*, and *B. hermsii* (unpublished) provides such an opportunity.

Here, we performed comparative genomics and genome-wide phylogenetic analyses to address the taxonomy of relapsing fever borreliae in a phylogenetic context, focusing on borreliae circulating in Africa.

## Materials and Methods

### Identification of orthologs

All chromosomal and plasmidic sequences were retrieved from GenBank for genomes: *B. crocidurae* (GenBank accession number CP003426), *B. duttonii* (CP000976), *B. recurrentis* (CP000993), *B. hispanica* (AYOU00000000), *Borrelia miyamotoi* (CP006647), *Borrelia persica* (AYOT00000000), *Borrelia hermsii* (CP000048), *Borrelia turicatae* (CP000049), *Borrelia bissettii* (CP002746), *Borrelia garinii* (CP003151), *Borrelia alzelii* (CP002933), and *Borrelia burgdorferi* (CP00222801). A bidirectional best hit (BBH) approach with an expect value cut-off of 10–3 was used to identify orthologous chromosomal genes; only genes having a similarity >30% and length coverage >70% were kept.

### Phylogenetic analysis

To compare the genomes of the relapsing fever borreliae, each set of orthologous chromosomal gene sequences was first aligned using MAFFT (17). Bayesian phylogenetic analyses were then performed using MRBAYES3.2.1 (18) with a GTR+ model of nucleotide substitution; sampling was performed through three independent runs (each having one cold chain and three heated chains), which were run for 1,000,000 generations or until the average standard deviation of split frequencies for the three runs was <0.01. Trees were sampled every 200 generations and the first one-fourth of the trees was discarded before a consensus tree was generated. Phylogenetic analysis of the telomere resolvase gene was conducted using a maximum likelihood approach with MEGA5 (19).

### Bayesian concordance analysis

The Bayesian concordance analysis was carried out with BUCKy 1.4.0 (20) using the phylogenetic trees generated in the Bayesian analysis with MRBAYES. For the discordance parameter α, we investigated three values representing the range of possible values: “0.01,” “1” (default value), and “2.” These values approximately translate into prior probabilities that two randomly sampled genes share the same tree of 0.99, 0.50, and 0.33, respectively. The final analysis used three runs of one million generations with eight chains (one cold chain and seven hot chains) and a discordance parameter α set to “1.”

### Selection analyses

To generate codon alignments, we first aligned each set of orthologous protein sequences with MAFFT (17). The amino acids were then replaced by the corresponding codons using PAL2NAL (21). Estimation of the non-synonymous (dN)/synonymous (dS) substitution rate ratio (ω) was performed using the maximum likelihood method implemented in the CODEML program from the PAML package (22) with the F3X4 model of codon frequencies. The ω ratio measures the selective pressure on coding sequences, with <1, =1, and >1 indicating negative purifying selection, neutral evolution, and positive diversifying selection, respectively. For each gene, the tree topology with the highest joint probably in the concordance analysis was used for these analyses and three sets of likelihood ratio tests were conducted to compare null models that do not allow ω > 1 (M1a, M7, and M8a) with models that do (M2a and M8). Significance was assessed by comparing twice the difference in likelihood between the models (2ΔL) to a χ^2^ distribution with one (M8a/M8) or two (M1a/M2 and M7/M8) degrees of freedom (22).

## Results

### Relapsing fever borreliae core and pan genome

**Figure 1:**
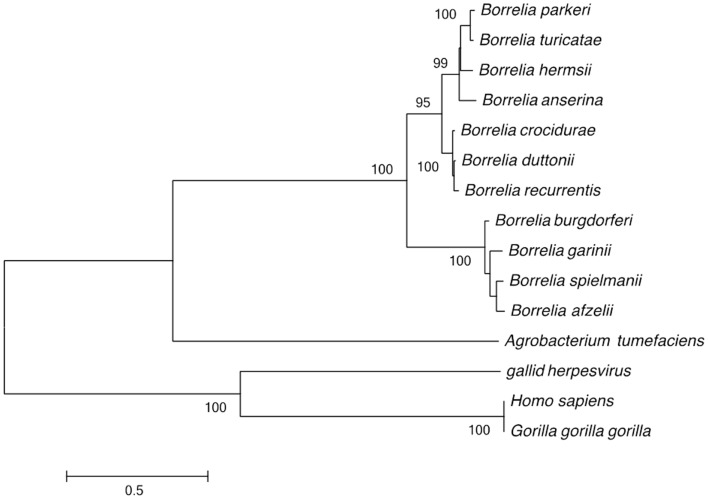
**Phylogenetic tree based on the *Borrelia* telomere resolvase gene sequence, separating the Lyme disease group from the recurrent fever group; and separating the Old World from the New World recurrent fever *Borrelia***. Only bootstrap values >80% are indicated at nodes. The bar indicates a 0.5% divergence in nucleotidic sequences.

The genomes of relapsing fever and Lyme disease borreliae include a linear chromosome, 1–15 linear plasmids, and 1–9 circular plasmids. This genome structure was compared to that of the 3,974 bacterial genomes described in the GOLD database. This analysis identified 755 fragmented genomes (19%), with 371 bacterial genomes consisting of one chromosome and one plasmid and 364 bacterial genomes including 3–11 DNA fragments (chromosome and plasmid). The remaining bacterial genomes were *Borrelia*, which are fragmented into 2–21 DNA fragments. In addition to *Borrelia*, linear chromosomes were only observed in *Streptomyces davawensis* and *Agrobacterium tumefaciens* (23, 24). The unusual linear DNA fragments of *Borrelia* have covalently closed telomeres (25). Phylogenetic analysis of the telomere resolvase gene grouped the *Borrelia* species into Lyme and relapsing fever groups. Furthermore, the *Borrelia* and the *A. tumefaciens* telomere resolvase genes clustered together and away from homologous gene in virus and eukaryotes (Figure 1).

*Borrelia hermsii* and *B. turicatae* chromosomes are collinear, whereas Old World borreliae genomes collinearity is disrupted by 1,134-bp insertion sequences (IS) IS605 OrfB (*B. duttonii, B. recurrentis*, and *B. crocidurae*) or 414-bp transposase IS200 (only in *B. crocidurae*). These IS are also carried by the plasmids of the three species and have high sequence identity [97–98%; Ref. (16)]. Chromosome collinearity is further disrupted by a 5-kbp duplication in *B. duttonii* (16). Also, genes rpsU, ftsK, and bacA that encode putative membrane proteins are duplicated in *B. recurrentis* (16). The main chromosomal differences between *B. duttonii, B. recurrentis*, and *B. crocidurae* exist in the 5′ regions of each chromosome; whereas in New World *B. hermsii* and *B. turicatae*, the 5′ regions are more conserved.

Comparing the gene content of *B. hermsii* against that of other *Borrelia* revealed five genes that were lost in some or all the non-*B. hermsii* borreliae: three hypothetical proteins (AAX17236, AAX17110, and AAX16935) that are unique to *B. hermsii*, one hypothetical protein (AAX16910) missing from relapsing fever borreliae but present in the Lyme group borreliae, and the glutathione peroxidase-like *Bsa*A gene (AAX17028) that is absent in non-*B. hermsii* borreliae but that has an ortholog in *Geobacillus thermoglucosidasius* and *Bacillus* species. A similar analysis using *B. turicatae* as a reference revealed one hypothetical protein (AAX17575) absent in non-*B. turicatae* borreliae, a second hypothetical protein (AAX18156) absent from relapsing fever borreliae but present in Lyme group borreliae, and the crossover junction endodeoxyribonuclease *Ruv*C (AAX17369) gene that is absent in non-*B. turicatae* borreliae but has an ortholog in *Clostridium acidurici*. Comparison of New World and Old World relapsing borreliae genomes identified two genes missing from the New World relapsing fever borreliae: RNA polymerase sigma-54 factor (ACH93394) and antigen P35-like protein (ACH92961). Conversely, one gene encoding the DNA repair protein RadA (AAX17754) is missing from the Old World relapsing fever borreliae genome.

**Figure 2:**
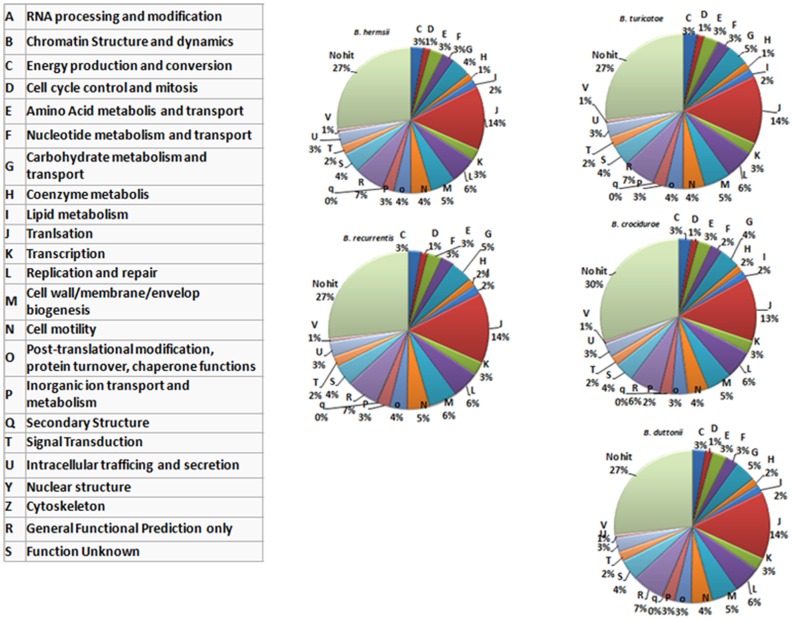
**Functional classification of the genes encoded by five recurrent fever *Borrelia* (chromosomal genes)**. The protein-coding sequences are classified according to categories determined in the cluster of orthologous group (COG) database, as indicated in the left-hand table.

In summary, the relapsing fever *Borrelia* pan genome includes 2,514 genes, and the number of protein coding genes (CDS) ranges from 990 in *B. recurrentis* to 2,140 in *B. crocidurae*. Of these, 933 (37.1%) represent the core genome and encode 788 core chromosome proteins and 145 core plasmid proteins. Interestingly, while the core genes represent <50% of the pan genome, they represent more than 81% of the genes classified into the cluster of orthologous group (COG) categories (Figure 2), consistent with the core genes representing conserved groups not only in *Borrelia* but also across other bacterial genera.

### Functional classification of the plasmidome

**Figure 3:**
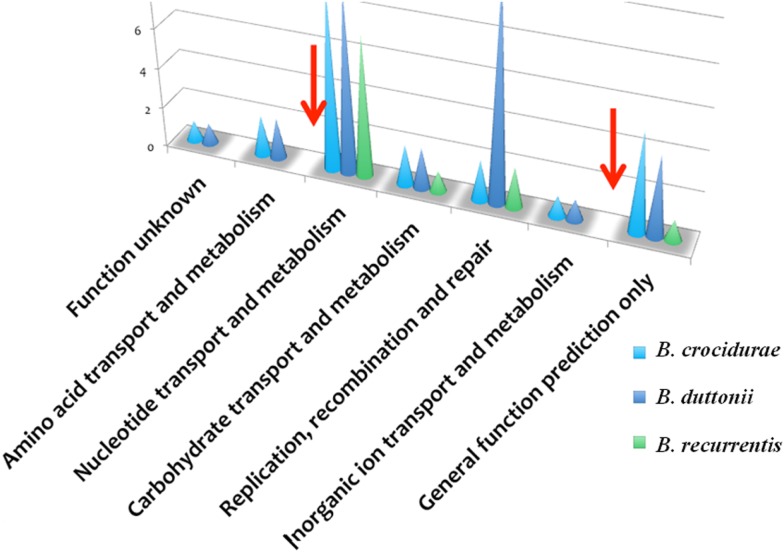
**Functional classification of plasmidic genes according to COG categories showing gene categories, which are missing in *B. duttonii* (dark blue symbol) and *B. crocidurae* (light blue symbol), compared to *B. recurrentis* (green symbol)**. Red arrows indicate known functions missing in *B. recurrentis*.

The *B. recurrentis, B. duttonii*, and *B. crocidurae* pan-plasmidome includes 706 genes, 162 of which are core plasmid genes, 166 genes being conserved in *B. crocidurae* and *B. recurrentis*, and 180 genes being conserved between *B. duttonii* and *B. recurrentis*. Interestingly, only 8–12% of the pan-plasmidome genes are assigned a functional category: this contrasts with the results obtained for the chromosomal genome, where 70–73% of the genes are assigned to a COG category (Figure 2), and is consistent with the plasmid genes evolving more rapidly than their chromosomal counterparts. Among the plasmidic genes, the most represented categories of genes are those involved in cell cycle control, cell division, chromosome partitioning translation while the least represented category is energy production and conversion. In *B. crocidurae* and *B. duttonii*, plasmidic genes could be assigned to 11 different categories and to 7 categories in *B. recurrentis* plasmidome. In particular, coenzyme transport and metabolism is missing from *B. recurrentis* (Figure 3). The *B. crocidurae* plasmidome encodes 607 proteins, 90% of which have homologs in the non-redundant database and an additional 9% are assigned putative function according to COG database. Some short genes are also present as remnants and thus represent potentially degraded genes. In the other species, plasmid sequence collinearity is more frequently interrupted by non-coding sequences and species-specific genes: accordingly, a low 42% density of CDs is due to large intergenic spacers. The five genomes encode glycoside hydrolase genes (CH23 and CH73) and glycosyl transferase family GT28 and GT51 (26) previously shown to be required for peptidoglycan synthesis (27), suggesting susceptibility to β-lactamines. Further comparisons against the Antibiotic Resistance Genes Database (28) indicate a complete absence of antibiotic resistance, in line with field observations that in Africa, antibiotics are regularly effective against relapsing fevers (29). Among studied species, only *B. crocidurae* contains one putative copy of clustered regularly interspaced short palindromic repeats (CRISPRs).

### Genome-wide phylogenetic analyses reveal a major evolutionary split

In order to further investigate the relationships between the *B. duttonii, B. recurrentis*, and *B. crocidurae* genomes, we used the 788 core chromosome genes and compared the phylogenetic signal obtained for each individual gene using a concordance analysis. This analysis reveals that the five genomes form two major clades: one containing *B. duttonii, B. recurrentis*, and *B. crocidurae* and second containing *B. hermsii* and *B. turicatae*; all 788 genes investigated support this clustering (concordance factor = 1).

**Figure 4:**
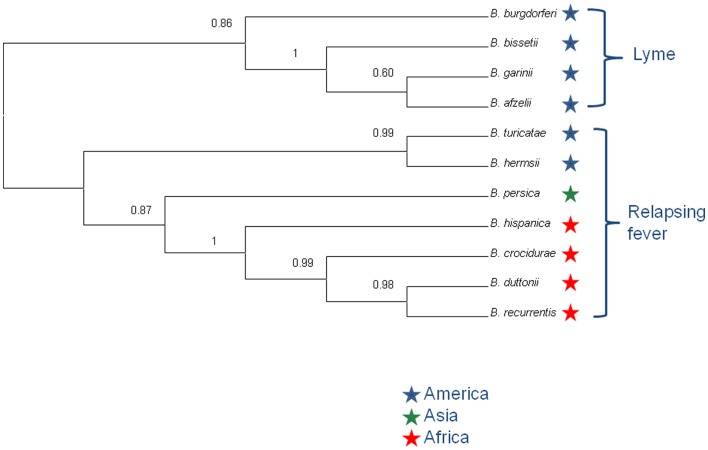
**Primary concordance analysis**. The tree shows the topology of *Borrelia* sp., grouped into the Lyme disease and relapsing fever group. Concordance factor values are indicated at nodes.

The concordance analysis also reveals a second well-supported split in the *B. duttonii, B. recurrentis*, and *B. crocidurae* clade, with the first two forming a group and the third one being an outgroup (concordance factor = 0.94). Consistent with this, only 16 of the 788 genes supported an alternative grouping for these three genomes (joint probability in the concordance analysis ≥0.5): 10 supported a *B. recurrentis*–*B. crocidurae* group and 6 a *B. duttonii*–*B. crocidurae* group. The tree topology was also conserved after including other relapsing and Lyme species (Figure 4). Further comparison of nucleotide sequences across all *Borrelia* genomes for the 788 core chromosome genes indicates an average nucleotide identity >99% between *B. duttonii, B. recurrentis*, and *B. crocidurae* (Table 1). Consistent with such a high identity at the nucleotide level, 173 proteins display identical amino acid sequences between *B. duttonii* and *B. recurrentis* and 108 between *B. crocidurae* and *B. duttonii*. Across these three species, 58 proteins display no sequence variation: those sequences notably include proteins involved in translation, ribosomal structure, and biogenesis (33%) as well as proteins involved in cell motility (10%); the remaining conserved proteins represent a large part (80%) of the COG functions.

**Table 1:** **Average nucleotide identity among *Borrelia* species**.

	*B. bissetii* (%)	*B. garinii* (%)	*B. afzelii* (%)	*B. burgdorferi* (%)	*B. turicatae* (%)	*B. hermsii* (%)	*B. crocidurae* (%)	*B. recurrentis* (%)	*B. duttonii* (%)	*B. persica* (%)	*B. hispanica* (%)
*B. hispanica* (%)	77.55	77.71	77.87	77.71	86	86	96.74	96.65	96.75	89.55	
*B. persica* (%)	77.63	77.64	77.63	77.59	85.9	86	89.36	89.32	89.37		
*B. duttonii* (%)	76	75.9	76.2	76	86.40	86.1	99	99.37			
*B. recurrentis* (%)	76	76	76.1	76.1	86.47	86.3	99				
*B. crocidurae* (%)	76	76	76.1	75.8	86.45	86.4					
*B. hermsii* (%)	76	76.1	76.2	76.1	91.20						
*B. turicatae* (%)	76.1	76.3	76.2	76.1							
*B. burgdorferi* (%)	94.8	92.5	92.8								
*B. afzelii* (%)	92.7	93.8									
*B. garinii* (%)	92.7										
*B. bissetii* (%)											

Consistent with their high nucleotide identity (86–99%), there was no significant difference in codon usage between the five relapsing fever borreliae, with the most frequently used codons in the chromosome (as measured by the relative synonymous codon usage or RSCU) being AGA, which encodes arginine (RSCU, 3.8), and TTA, which encodes leucine (RSCU, 2.6). Similarly, the least used codons are CGG and CGC that encode arginine and CTG, which encodes leucine.

### Positive diversifying selection contributed to the evolution of the *Borrelia* genomes

To investigate how natural selection contributed to the evolution of the five relapsing fever borreliae genomes, we performed a genome-wide analysis of positive selection: significant evidence of positive diversifying selection was observed for 55 of the 788 core chromosome genes (α ≤ 0 with at least two of the three methods used; Table 2). Of these 55 genes, 8 (14.5%) are involved in translation, ribosomal structure, and biogenesis, 5 (9%) in replication, recombination, and repair, and 4 (7.2%) in cell wall biogenesis, cell motility, and signal transduction mechanisms.

**Table 2:** **Analysis of positive selection in five *Borrelia* species**.

Locus	2*Delta-likelihood			Statistical significance		Function
	M1a vs. M2a	M7 vs. M8	M8 vs. M8a	No. of methods	Level	
1	47.95	52.16	47.20	3	0.01	Chemotaxis protein methyltransferase 287 aa
2	24.64	27.71	26.94			Chromate transport protein, putative 192 aa
3	19.87	24.40	19.95			Uncharacterized conserved protein 272 aa
4	13.59	16.44	14.91			Conserved hypothetical integral membrane protein 293 aa
5	13.43	21.33	12.10			Uncharacterized conserved protein 302 aa
6	13.10	15.23	14.39			Carboxypeptidase, putative 280 aa
7	12.90	14.74	13.82			Phosphomevalonate kinase, putative 313 aa
8	12.44	16.77	14.02			Chemotaxis response regulator 136 aa
9	11.42	16.25	12.59			Uncharacterized conserved protein 260 aa
10	10.86	11.04	10.05			*S*-adenosylmethionine synthetase 389 aa
11	10.52	13.30	12.23			UDP-*N*-acetylglucosamine–*N*-acetylmuramyl-(pentapeptide)
12	10.21	14.66	10.12			Uncharacterized conserved protein 273 aa
13	9.68	13.29	8.37			ATPase involved in DNA repair 528 aa
14	9.69	11.77	9.78			Holo-acyl-carrier protein synthase 129 aa
15	8.67	19.49	9.99	3	0.05	CTP synthase 551 aa
16	7.54	13.99	7.68			Chemotaxis protein CheD 167 aa
17	7.32	12.10	7.92			l-Lactate dehydrogenase 320 aa
18	7.01	11.35	5.23			Uncharacterized conserved protein 422 aa
19	6.67	10.71	8.80			tRNA pseudouridine 55 synthase 281 aa
20	6.65	7.91	7.76			HemN-related protein 379 aa
21	6.52	16.42	6.70			Uncharacterized conserved protein 257 aa
22	6.09	11.58	8.74			Exported protein 357 aa
23	6.00	9.77	6.50			Uncharacterized conserved protein 313 aa
24	6.33	7.93	6.36			Flagellar protein 143 aa
25	8.71	6.98	4.16			Signal recognition particle protein 449 aa
26	50.59	18.29	3.27	2	0.01	Hypothetical protein BDU_209 731 aa
27	5.94	13.88	7.67			Uncharacterized conserved protein 334 aa
28	5.47	11.08	7.45			Methyl-accepting chemotaxis protein 391 aa
29	4.48	9.35	7.18			DNA gyrase, subunit A 815 aa
30	5.82	9.27	6.95			Flagellar motor rotation protein B 262 aa
31	5.74	11.53	6.49	2	0.05	Uncharacterized conserved protein 183 aa
32	5.80	15.34	6.19			Uncharacterized conserved protein 477 aa
33	4.48	6.74	6.13			Uncharacterized conserved protein
34	5.96	12.34	6.10			Lysyl-tRNA synthetase 532 aa
35	4.08	9.73	6.08			ATP-dependent Clp protease, subunit X 429 aa
36	5.98	14.59	6.05			1-Acyl-sn-glycerol-3-phosphate acyltransferase 250 aa
37	5.67	9.23	5.66			30S ribosomal protein S5 164 aa
38	5.46	10.80	5.43			Conserved hypothetical GTP-binding protein 368 aa
39	5.23	20.06	5.40			Trigger factor 448 aa
40	4.52	8.65	5.21			Exodeoxyribonuclease V, gamma chain 1070 aa
41	5.16	13.81	5.17			Hemolysin 259 aa
42	4.49	7.03	4.51			Uncharacterized conserved protein 143 aa
43	4.01	15.65	4.40			Translation elongation factor G 674 aa
44	4.49	15.41	3.98			p-512 protein 2361 aa
45	3.20	7.28	6.39			Flagellar protein 389 aa
46	2.95	6.81	5.93			Glycerol-3-phosphate dehydrogenase, NAD(P) 350 aa
47	3.60	8.25	5.64			Hypothetical protein BDU_543 361 aa
48	2.89	7.35	5.60			Phenylalanyl-tRNA synthetase alpha chain 515 aa
49	3.25	6.82	5.49			Uncharacterized conserved protein 492 aa
50	3.77	7.18	5.17			Uncharacterized conserved protein 359 aa
51	3.05	11.02	5.07			DNA helicase 698 aa
52	1.14	6.49	3.94			Purine-binding chemotaxis protein CheW 465 aa
53	3.07	9.44	3.91			tRNA (5-methylaminomethyl-2-thiouridylate)-methyltransferase 354 aa
54	2.84	6.41	3.90			3-Methyladenine DNA glycosylase 196 aa
55	3.81	11.72	3.86			Uncharacterized conserved protein 270 aa

*This analysis indicates that 55 chromosomal genes are under positive selection*.

## Discussion

Thanks to the sequencing of several African relapsing fever borreliae (14–16), it is now possible to assess relationships between African and American relapsing fever borreliae. Interestingly, all recurrent fever *Borrelia* shares an overall genome architecture with Lyme disease group borreliae that is unique, in that they exhibit a massively linear genome. This unique topology is maintained by one plasmidic copy of telomere resolvase, an enzyme rarely found in bacteria. *Borrelia* telomere resolvase is related to that of *A. tumefaciens*, a plant pathogen unrelated to *Borrelia*.

Whole chromosome comparisons across American and African relapsing fever borreliae show extensive conservation of gene content and gene order, only disrupted by missing genes in one group. As for genes shared between *B. duttonii, B. recurrentis*, and *B. crocidurae, B. turicatae*, and *B. hermsii*, the average nucleotide identity is ~86%. The high number of impaired genes in *B. recurrentis* might be indicative of a decaying genome (16). In bacteria, RecA and RadA proteins play a critical role in DNA repair processes (30): both genes are present in the New World relapsing fever borreliae that also have impaired genes. In contrast, *B. crocidurae* and *B. duttonii* encode RecA but not RadA and have a lower number of impaired genes, while *B. recurrentis* lacks both proteins and contains many impaired genes. The accurate pan-genome size is probably bigger than the one we estimated.

Close relatedness between *Borrelia* species is further reflected in their gene content as they share >90% of their genes. Similarly, a high level of functional conservation was observed, as many orthologous proteins share high sequence identity. While the chromosomes have the same codon usage, different codon usage distinguishes the chromosome and the plasmids in the same species. Indeed, the majority of housekeeping genes are chromosomal whereas the plasmids mostly encode surface proteins responsible for antigenic variability and host-interactions. It became apparent that some plasmid genes code for unique functions not represented in the chromosome, in addition to some function that are shared with chromosomal genes. Plasmids are frequently subject to genomic rearrangement and, not surprisingly, borreliae plasmids are not totally collinear and conserved in terms of gene content and orientation but they still share syntenic sequences representing the core plasmid. As for the African relapsing fever borreliae chromosome, 90% of its gene content is included in the core genome and is thus common to the three sequenced genomes; the remaining 10% represent species-specific genes that lie in the 5′ extremities. Interestingly, the variable sequences in these 5′ extremities are related to plasmid sequences, a phenomenon already observed for the *B. burgdorferi* chromosome (31).

Together with variation in plasmid content, positive selection represents one of the main molecular mechanisms contributing to vector–host adaptation. Our study revealed that 55 genes were the targets of positive diversifying selection during the evolution of the recurrent fever borreliae. Although these genes are randomly distributed in the chromosome, they code for proteins involved in translation, ribosomal structure and biogenesis, replication, recombination and repair, cell membrane biogenesis, cell motility, and signal transduction: these mechanisms are related to host interaction and might thus be important factors to restrict the habitats of *Borrelia* species to specific niche (i.e., host and vector adaptation).

## Conclusion

Current taxonomy of the relapsing fever borreliae derives from geographical and tick-specific relationships comforted by analyses of single genes or of several concatenated genes (32). The coexistence of different species in the same geographic region questioned the accuracy of this conventional classification and here we aimed to gain a more accurate picture by using a genome-wide phylogenetic approach, in line with recent recommendations to incorporate genome sequence data into classification of bacteria (33). This analysis grouped the relapsing fever borreliae into a clade A that includes Old World *B. hispanica, B. recurrentis, B. crocidurae*, and *B. duttonii* and a clade B that includes New World *B. turicatae* and *B. hermsii*. This division is supported by the 788 genes used. The concordance analysis also strongly suggests that *B. duttonii* and *B. recurrentis* are closely related and form a clade that has *B. crocidurae* as an outgroup, strengthening a previous hypothesis that *B. recurrentis* is a subset of *B. duttonii* (16). Accordingly, the average nucleotide identity is 99% between *B. crocidurae, B. duttonii*, and *B. recurrentis*, 96% between *B. hispanica* and (*B. crocidurae, B. duttonii*, and *B. recurrentis*), and 91% between *B. turicatae* and *B. hermsii*, while the ANI between the two groups is at 86%. It was suggested that average nucleotide identity values of ~94% correspond to the cut-off for bacterial species definition (34), so that the average nucleotide identity for *B. duttonii, B. crocidurae*, and *B. recurrentis* would be well above that level and the three Old World species would thus be ecotypes of the same genomospecies. These data suggest updating the long-term taxonomy of these borreliae, in delineating a new species *Borrelia africana*, comprising of three subspecies *crocidurae, duttonii*, and *recurrentis*. Also, data here presented allow refining molecular tests for the accurate detection and identification of relapsing fever borreliae, in a point-of-care format (35).

## Author Contributions

Pierre Pontarotti and Michel Drancourt designed the study. Haitham Elbir, Laurent Abi-Rached, and Niyaz Yoosuf performed analyses. Haitham Elbir, Laurent Abi-Rached, Pierre Pontarotti, and Michel Drancourt analyzed the data and prepared the manuscript. All authors read and approved the final manuscript.

## Conflict of Interest Statement

The authors declare that the research was conducted in the absence of any commercial or financial relationships that could be construed as a potential conflict of interest.
